# The Need to Incorporate Post-Acute Care Entities Into the National Disaster Medical System

**DOI:** 10.1016/j.acepjo.2025.100237

**Published:** 2025-08-19

**Authors:** Adeteju Adeniji Ajibade, Reena Sethi, Clark J. Lee, Emily R. Post, Shelley O. Meeks, Tahroma Alligood, Kaitlin Rainwater-Lovett, Michelle M. Kimball, Ryan Leone, Michael Zanker, Jeffrey D. Freeman, Thomas D. Kirsch

**Affiliations:** 1 The National Center for Disaster Medicine and Public Health (NCDMPH), Uniformed Services University of the Health Sciences (USU), Bethesda, Maryland, USA; 2 The Henry M. Jackson Foundation for the Advancement of Military Medicine, Inc, Bethesda, Maryland, USA; 3 Guidehouse Inc, McLean, Virginia, USA; 4 Columbia University Vagelos College of Physicians and Surgeons, New York, New York, USA; 5 Administration for Strategic Preparedness and Response (ASPR), U.S. Department of Health and Human Services, Washington, District of Columbia, USA

**Keywords:** healthcare preparedness, medical surge, disaster medicine, post-acute care, National Disaster Medical System

## Abstract

The National Disaster Medical System (NDMS) is critical to healthcare preparedness and response in the United States but currently has limited capacity for handling large-scale mass-casualty surge events. Crises, such as the COVID-19 pandemic, have shown how quickly the US healthcare system can become overwhelmed, necessitating novel solutions for handling a large-scale influx of patients. This paper proposes the inclusion of post-acute care (PAC) entities, which provide long-term care rehabilitation and short-stay subacute rehabilitation support rather than acute or critical care, into the NDMS as one solution to increase its capacity during mass-casualty surges. By reviewing the unique capabilities and functions of PAC entities and considering evidence of their role in national emergencies, this paper demonstrates that PAC entities are well positioned to support the wider healthcare system in managing a mass influx of patients. We base this assertion on 4 points: (1) the support role PAC entities traditionally play for over-capacity hospitals by providing extra space for stabilized patients, (2) PAC entities’ capacity to adapt their functions beyond PAC to support overall community response, (3) recent federal emergency preparedness requirement changes that increase PAC entities’ disaster response capabilities, and (4) interim outcomes of NDMS efforts toward NDMS inclusion of PAC entities. We conclude by outlining challenges to integrate PAC entities into the NDMS and highlighting ongoing work by the congressionally directed NDMS Pilot Program, which is designed to increase capacity across the system. However, challenges such as limited staffed bed capacity at PAC entities, reduced availability of emergency medical service ambulances, and ongoing staffing shortages will need to be addressed.

## 1 Introduction

The National Disaster Medical System (NDMS) was established in 1984 to provide healthcare for combat casualties resulting from an overseas conflict and to supplement state and local medical services during domestic emergencies.^1^^,^^2^ The system includes deployable medical teams and equipment to assist in an initial disaster response, patient transportation capabilities, and a national network of hospitals to provide definitive care for both disaster and combat casualty patients.^3^ Despite these assets, concerns that the current NDMS is insufficient for a large-scale medical surge event^4^ were reinforced by the COVID-19 pandemic, which stressed the US healthcare system and infrastructure.^5^ This suggests that opening additional avenues for reducing the requirements of acute care hospitals will create medical and public health gains for the nation. Currently, NDMS patient care guidelines end at the hospital and post-acute care (PAC) entities are not incorporated into the definitive care pathway. However, patients recovering from disaster events served by NDMS often require recovery and rehabilitation services offered by PAC entities. One potential means to augment the surge capacity of the NDMS and strengthen the US healthcare system’s ability to respond to large-scale events is to incorporate PAC entities into the NDMS network.

### 1.1 PAC Entities

PAC consists of services to individuals that aid their return to their communities after their conditions have stabilized following acute hospitalization.^6^ PAC services include medical treatment, nursing care, rehabilitation, and residential care.^6^^,^^7^ There are currently 4 types of PAC entities in the United States: inpatient rehabilitation, long-term care, skilled nursing, and home health care. Although each PAC entity type provides different services (see Table 1),^8^, ^9^, ^10^, ^11^ all PAC entities share 5 essential characteristics or goals:

1. Service recipients cannot need acute long-term hospitalization or premature placement in a long-term care facility.
2. Services provided are based on a comprehensive assessment of the care recipient and the resultant individualized treatment plan for the recipient.
3. Services should strengthen recipients’ autonomous living abilities with the aim of transitioning to a lower level of care with the goal of returning home independently.
4. Services last a minimum of 2 weeks.
5. Services include all treatment areas and incorporate individual assessment mechanisms, individual case history records, and case history sharing procedures.^6^

**Table 1:** Post-acute care entity types.

Entity	Entity type	Operational services provided	Patient length of stay	Quantity
Inpatient rehabilitation facilities and units	Inpatient—within and independent of hospitals	Minimum of 3 h of daily rehabilitation following an acute illness or injury.	Approximately 2 wk	∼1211^8^
Long-term care facilities	Inpatient—within and independent of hospitals	Treat stable patients with multiple comorbidities or care needs who require assistance from the medical team and also provide primary care, nursing, specialty care, and drug therapy.	Several mo to y	∼65,600^9^
Skilled nursing facilities	Inpatient—nursing homes	Provide physician’s rounds, rehabilitation, nutrition care, and skilled nursing measures such as drug treatment, wound treatment, and ventilator care.	Approximately 6 wk	∼15,300^10^
Home health agencies	Home health	Provide skilled nursing care, physical therapy, functional and language therapy, social work services, and other healthcare services.	Several mo to y	∼11,400^11^

PAC entities share several capabilities that have been deployed during past responses to disasters and other local and regional medical surge events that make them attractive resources for inclusion in the NDMS.^12^^,^^13^ More specifically, PAC entities can help nearby acute care hospitals alleviate overcrowding and adapt to support local response efforts during large-scale disasters in their communities. PAC entities nationwide are also better prepared to support local and regional responses to mass-casualty events as a result of recent changes in the Centers for Medicare and Medicaid Services’ emergency preparedness requirements that now include PAC entities, as described below.^14^

### 1.2 PAC Entities as “Pressure Release Valves” for Hospitals During Past Large-Scale Surge Events

PAC entities have played an underappreciated role in expanding the healthcare system’s surge capacity during crises. For example, after the 1994 Northridge earthquake near Los Angeles, California, hospitals and PAC facilities that were damaged or over-capacity requested patient transfers to undamaged PAC facilities.^12^ This allowed hospitals to focus on acute earthquake injuries, minimizing deaths from the earthquake. More recently, during Hurricane Irma in 2017, PAC entities alleviated the burden on local hospitals by accepting an increased volume of stabilized patients, making room at hospitals for those injured by the storm. PAC entities also prepared for a potential patient surge by temporarily increasing nursing staff through agencies, stockpiling resources, and preparing the entity to withstand the storm, enabling the acceptance of an increased volume of patients who no longer required inpatient level of care at hospitals.^15^

During the COVID-19 pandemic, PAC entities played a role in relieving hospitals during periods of high patient volume. The State of Connecticut opened specialized PAC facilities for recovering COVID-19 patients to be transferred to receive rehabilitation services, specialty care, and extended stay services.^16^ These “COVID-19 only” facilities used existing spaces that previously or currently operated as PAC facilities but had been vacated of patients. Surviving patients transitioned home to continue their recovery with the support of home health services.

In 2020, the Montefiore Health System in New York City increased its capacity for COVID-19 patients by expanding the number of PAC entities in its healthcare network.^13^ Montefiore’s patient transition process was adapted to easily assess patients for PAC needs, and in-network PAC entities altered their admissions process and requirements to include recovering COVID-19 patients. Montefiore further collaborated with local home health agencies to support discharged COVID-19 patients who required home oxygen. As a result, Montefiore increased patient capacity at both its hospitals and PAC entities.

These examples illustrate how PAC entities are valuable disaster response partners during a medical surge event. Given their routine role of providing care to individuals no longer needing long-term hospitalization, PAC entities can be critical partners for expanding bed availability to patients who no longer require inpatient level of care from hospitals.^17^ Through reverse triage, hospitals can identify patients most appropriate for disposition to PAC entities to ensure balanced patient load.^18^^,^^19^ PAC entities thus function as “pressure release valves” for hospitals reaching the limits of their surge capacity during a crisis.

### 1.3 Adaptability of PAC Entities to Directly Support Community Responses to Large-Scale Surge Events

PAC entities have been adapted for functions beyond providing spaces for local hospitals to offload recovering COVID-19 patients. For example, PAC entities were key partners in a number of alternate care sites staged to manage rapid increases in COVID-19 patients in local and regional health systems around the United States.^20^ In Boston, the local government, major healthcare system, and military partners collaborated to quickly stage a 1000-bed field hospital that included 500 respite beds for individuals with COVID-19 who only required isolation and 500 PAC beds for COVID-19 patients from hospitals and outpatient settings who also required transitional or respite care.^21^ The 500 PAC beds were developed, resourced, and managed by a local multisite healthcare system that included a PAC network, community hospitals, and large academic medical centers. This example of a cross-sector response to a local medical surge event involving isolation care illustrates how PAC entities could be leveraged at scale during an NDMS activation in response to a nationwide surge event while also addressing routine patient needs. Furthermore, should federal emergency preparedness decision-makers continue their shift away from federally managed alternate care sites, inclusion of PAC entities in the healthcare system may help fill this need.

Additionally, PAC entities can vary in the type of care provided, bolstering their adaptability and utility in disaster response. For example, although inpatient rehabilitation facilities, long-term care facilities, and skilled nursing facilities are pre-existing care centers that can be used in surges, home health agencies are able to turn residential homes into supplemental capacity. Thus, with potential guidance from locally available paramedicine programs or telemedicine solutions, home health agencies and patient homes can expand capacity among the larger PAC entity system to further relieve the healthcare system and ensure that the most acute patients are receiving hospital beds.

### 1.4 Recent Federal Emergency Preparedness Requirements Have Primed Post-Acute Care Entities to Support Local Responses to Large-Scale Surge Events

Incorporating PAC entities into the NDMS has become more feasible in the past decade due to federal regulatory developments governing how PAC entities in the United States prepare for emergencies. Following the identification of response vulnerabilities in various types of healthcare facilities exposed during earlier disasters, particularly hurricanes Katrina and Sandy, the Centers for Medicare and Medicaid Services has required PAC entities since 2016 “to plan adequately for both natural and man-made disasters, and coordinate with federal, state, tribal, regional, and local emergency preparedness systems” as a condition of participation in Medicare and Medicaid.^14^^,^^17^^,^^22^ Under these national requirements, PAC entities must (1) perform “all-hazards” risk assessments to establish entity-specific emergency plans; (2) develop and implement policies and procedures to execute these emergency plans and address risks identified from the aforementioned assessments; (3) develop and maintain emergency preparedness communication plans to protect the health and safety of their clients during disasters while coordinating with partners in the entity, nearby healthcare providers, and state and local public health and emergency management authorities; and (4) develop and maintain emergency preparedness training and testing programs for their staff.^14^^,^^17^^,^^22^ As a result of this rule, advanced planning during Hurricanes Florence and Michael led some facilities to stock a full week’s worth of supplies and fuel.^23^ The emergency preparedness activities recently undertaken by PAC entities to comply with these federal requirements suggest that PAC entities are better prepared to support their local healthcare system and communities during a disaster or other large-scale surge event.

## 2 Discussion

### 2.1 Why and How Post-Acute Entities Should be Included in the NDMS

The COVID-19 pandemic repeatedly illustrated how quickly the US healthcare system can become overwhelmed when it does not have sufficient support for its clinical and emergency response operations. However, this and other past crises have encouraged the healthcare sector to find novel solutions for mass-casualty events,^24^ such as using PAC entities to alleviate patient surge in local hospitals and health systems.^13^^,^^21^ Securing the participation of PAC entities in NDMS planning and response can increase the ability of both the NDMS and broader US healthcare system to effectively respond to a variety of large-scale surge events, including domestic disasters and overseas military conflicts resulting in large numbers of casualties.

However, there will be challenges associated with incorporating these PAC entities into the NDMS. Limited staffed bed capacity at PAC entities, availability of emergency medical service ambulances, and staffing shortages will need to be addressed. PAC staffing assets are not the same as they were prior to the COVID-19 pandemic.^25^ There are nursing and physician shortages across the larger healthcare system in addition to PACs that require additional planning to optimize staffing models. Developing a robust regional PAC network would be a major step toward patient load balancing among these facilities during crises, in addition to interstate licensing for healthcare providers.^26^ Including PAC entities will also require coordinating healthcare patient tracking systems and entities’ bed availability from point of injury to discharge from a PAC entity to manage patient surge. Local and national NDMS leadership will need to expand and sustain engagement with PAC entities, secure their participation, and train and educate their staff and administration on their role within the NDMS network. This will entail inviting PAC entities to join NDMS through a signed Memorandum of Agreement (MOA) and including PAC entity leadership in the higher-level planning of patient surge management. Training guides need to be developed, and PAC entities should perform exercises to practice procedures and ensure staff have full awareness of expectations during a catastrophic event. This will require new funding and capabilities for the NDMS itself and possibly legislative, regulatory, or policy changes to ensure those capabilities are met. For example, the process for reimbursement during a large-scale surge event and a plan to sustain multiyear funding for the NDMS to guarantee it reaches its fullest capabilities will need to be delineated. By including PAC entities in the NDMS definitive care network, improved planning efforts and coordination will help to maintain positive relationships among provider entities.

Although the NDMS is designed to be activated both in the event of a domestic disaster and a large-scale overseas combat casualty scenario, PAC entities would likely respond differently to various types of events. For example, during a domestic disaster response, entities in the affected area may not be available to augment patient capacity, whereas during a combat casualty situation, entities may receive patients who have significantly different needs than the patients typically served by the entities (eg, a service member recovering from combat injuries and multiple surgeries versus a traditional PAC patient or patients requiring isolated care during a pandemic). Additionally, PAC entity patients who are offloaded or have their care delayed as a result of PAC entity utilization during a large-scale surge event need a clear destination for their continued care and recovery. Early discharge from PAC facilities can result in a more extensive need for home health skilled nursing and therapy care if patients are not fully recovered. Continuity of patient care can be compromised in this environment. Furthermore, PAC facilities receive patients who are at risk of rehospitalization,^27^ which is associated with high morbidity and mortality, so securing transportation may be difficult.

To make sure that PAC entities are most effectively employed in disaster scenarios, consideration must also be given to the level of skilled care that they can provide. In 2023, referral volumes from hospitals to skilled nursing facilities and home health agencies increased 10% and 11%, respectively, from the prior year, and the relative severity of hospital patients increased by 6% since 2019.^28^ This surge in referrals and rising severity of hospital patients means that PAC entities may already be receiving a higher volume of patients who require more care than previous patients. Home health referral denial rates also spiked by 40% in this period, indicating that PAC entities may be currently unwilling or unable to offer care to larger quantities of higher-severity patients.^28^ As a result, work must begin now to consider how PAC entities can be upskilled before times of crisis. PAC providers should be offered training opportunities to improve their ability to manage complex patient needs related to combat casualties, clinical staff leaders from hospitals should prepare to serve as augmenters to PAC entities that begin accommodating NDMS patients, and supply gaps that would otherwise limit PAC entities from offering care should be identified and rectified. On top of this, federal policy changes should be built in, such as expanding eligibility for waivers to Medicare’s rule that it will only cover skilled nursing facility stays after a patient has already been hospitalized for 3 days,^29^^,^^30^ and PAC entity rules should change to prevent the protocol-based rejection of patients with higher care needs.

Outside of upskilling PAC entities, capacity can be increased by preemptively improving transfers of care. By identifying barriers to sending patients to PAC entities that exist in “blue skies” environments, hospitals can rapidly discharge less acute patients to PAC entities and maintain higher standing bed capacity before disaster strikes, instead of beginning the administrative, logistic, and clinical steps needed to move these patients during a crisis. Therefore, obtaining PAC buy-in for disaster response might not just be having agreements signed that are enacted during contingencies, but rather providing ongoing support to optimize the efficiency of routine hospital-to-PAC discharges. One promising mechanism for improving such transfers is through Medical Operations Coordination Centers and related approaches developed across the United States over the years to support patient load balancing and patient transfer management between healthcare facilities at the regional, state, and federal levels during steady-state and crisis scenarios.^31^, ^32^, ^33^

### 2.2 NDMS Pilot Program PAC Entity Inclusion Progress and Outcomes

Fortunately, efforts are underway to better understand how a PAC entity can assist in an NDMS activation by accounting for bed capacity, supplies, planning, and coordination between state and local facilities, and communication within partnering entities. Since 2020, the US Department of Defense, in collaboration with the Departments of Health and Human Services, Homeland Security, Transportation, and Veterans Affairs, has led a congressionally directed NDMS Pilot Program (Pilot) to expand the NDMS’s medical surge capability and capacity and to improve interoperability between the NDMS’s military and civilian partners.^34^, ^35^, ^36^ The Pilot has collected information and developed insights on the need to include PAC entities into the NDMS, and the roles these care providers would have during an NDMS activation.^37^, ^38^, ^39^ The Pilot is also working with its federal and Pilot site partners to identify and formalize the processes for including PAC providers in the NDMS definitive care network.

A Pilot-sponsored project concluded in May 2024 included an evaluation of the PAC entities within a 22-county Trauma Service Area-P in Southwest Texas administered by the Southwest Texas Regional Advisory Council. All PAC entities in the region, inclusive of inpatient rehabilitation facilities, long-term acute care hospitals, and skilled nursing facilities, were assessed for clinical capabilities, care coordination, capacity, and willingness to participate and sign an MOA to partner with NDMS (Table 2).

**Table 2:** Process discoveries from the 2024 NDMS Pilot study of PAC entities in Southwest Texas.

Outreach approach	Data and technology	Care coordination	Home health and hospice	Additional needs
• Leveraging corporate and regional leadership contacts is vital for foundational engagement, participation, and creating an efficient communication process. • Engage state associations representing PAC entities. • Engage key local stakeholders. • Prioritize engagement with high-volume, high-performing, and geographically appropriate entities.	• Appropriate documentation must follow the patient through the care continuum. • PAC entity bed availability is a point-in-time metric and can change daily.	• National emergency waivers are required to allow for timely admissions and smooth transitions. • Waivers must be inclusive of TRICARE requirements.	• Investigation is needed to define the end of care and regulatory requirements associated with home health and hospice. • Confirm existing outpatient post-acute network structure and process and gaps for home health and Hospice with TRICARE and VA care coordination.	• The MOA is currently geared to acute entities; language should be updated for PAC inclusion. • Post-acute entities require sustained NDMS updates and education. • Ongoing coordination with TRICARE’s managed service provider (Humana Military) is important to ensure efficient delivery of care.

NDMS, National Disaster Medical System; MOA, Memorandum of Agreement; PAC, post-acute care.

Once PAC entity data were collected, a summary tool was created with an overview of clinical capabilities, bed capacity, and interest in participation in the NDMS. This tool was then used during a tabletop exercise to identify placement potential for combat casualties at a PAC entity location. Lastly, the entities that acknowledged intent to participate in the NDMS received outreach to complete an MOA and reviewed PAC roles and responsibilities as described in a regional charter and detailed PAC entity standard operating procedure. PAC’s roles and responsibilities were tested in a full-scale interagency regional functional exercise. Project findings guided subsequent Pilot activities by identifying additional NDMS development opportunities and potential gaps requiring further evaluation (Table 3).

**Table 3:** Findings, recommendations, and pilot activities for process improvement.

Findings
1. Identification of corporate and regional leadership contacts of post-acute entities, which are necessary for successful development of a PAC network. 2. National emergency waivers and clear understanding of application of these waivers are required to allow for timely admissions and smooth patient care transitions. 3. The PAC patient coordination process is directly impacted by other patient care settings, and care coordination roles and responsibilities are shared across stakeholders. 4. Military case manager roles and responsibilities are not well defined. 5. PAC case managers are dependent on acute, military, and other care setting case managers to effectively coordinate care needs of the patient; adequate governance, personnel, and IT platforms to enable information-sharing and open lines of communication between case managers are required to support the continuity of the patient journey and maximize facility and system capacity.

Recommendations

1. NDMS should continue to expand upon recent distributed community network MOA work to include language more tailored to post-acute levels of care. 2. Fully define the end of the episode of care and regulatory requirements associated with home health and hospice. 3. Implementing consistent, clear SOPs with detailed roles and responsibilities at each level of response for each stakeholder is crucial to ensure warm handoffs. 4. Develop, implement, and incorporate DoD policy to direct consistent roles and responsibilities among and across services and DHA. 5. To ensure care continuity and tracking (via JPATS, TRACES, Pulsara, EMRs, etc), it is necessary to test among systems the flow of specific patient data required, including testing during removal or loss of wristbands or other physical tracking methods, from patient arrival to the end of the episode of care.

Ongoing pilot activities

1. A legal, regulatory & policy review of the NDMS, including mechanisms for CMS waivers applicable to acute and PAC facilities during large-scale disasters. 2. Understanding the Military Health System as it relates to the case management of service members during a large-scale combat casualty event. 3. Engaging new partners to create a federal-level NDMS PAC network that can provide leadership and support to new civilian partners. 4. Four pilot sites (Denver, Omaha, Sacramento, and San Antonio) maintain a post-acute care and nonhospital master list of participating and potential organizations, information session materials, facility activation plans, and healthcare communication engagement strategies. 5. Understanding regional Medical Operations Coordination Centers and other existing approaches for supporting regional patient load balancing and patient transfer management between healthcare facilities (including PAC entities) during a large-scale combat casualty event or other nationwide medical surge event.

CMS, Centers for Medicare & Medicaid Services; DHA, Defense Health Agency; DoD, Department of Defense; EMR, electronic medical records; JPATS, Joint Patient Assessment and Tracking System; MOA, Memorandum of Agreement; NDMS, National Disaster Medical System; PAC, post-acute care; SOP, standard operating procedure; TRACES, Transportation and Controlled Environment System.

## 3 Conclusion

Integrating PAC entities into the NDMS is critical for increasing the capacity of the healthcare system to respond to a large-scale disaster. Previous disaster and patient surge situations have demonstrated that the integration of PAC entities can effectively augment patient capacity and improve coordination among acute care and PAC entities. Although the role of PACs may change during a large-scale combat operation, PACs have demonstrated the ability to adjust to challenges arising from disasters. The Pilot is actively advancing these efforts in coordination with federal, state, and local partners. The future activities of the Pilot program include development and execution of plans to load balance patients across PAC entities from patient reception sites, decompress healthcare facilities to ensure appropriate medical care and resources for patients, and continue encouraging integration of PAC facilities into the NDMS.

## Author Contributions

AAA: conceptualization, investigation, writing—original draft, and writing—review and editing.

RS: conceptualization, writing—original draft, writing—reviewing and editing, and supervision.

CJL: conceptualization, writing—original draft, and writing—review and editing.

ERP: writing—original draft and writing—review and editing.

SOM: conceptualization, investigation, and writing—review and editing.

TA: conceptualization, investigation, and writing—review and editing.

KRL: writing—review and editing and supervision.

MMK: conceptualization, resources, and writing—review and editing.

RL: original draft and writing—review and editing.

MZ: writing—review and editing.

JDF: writing—review and editing.

TDK: conceptualization, writing—original draft, writing—review and editing, and supervision.

## Funding and Support

The authors received an award from The Office of the Assistant Secretary of Defense for Health Affairs (awarding office), 77000 Arlington Blvd., Suite 5101, Falls Church, VA 22042-5101: HU00012420023, and the Uniformed Services University of the Health Sciences (USUHS), 4301 Jones Bridge Road, Bethesda, MD 20814 was the administering office.

This project is sponsored by 10.13039/100007188USUHS; however, the information or content and conclusions reported in this work do not necessarily represent the official position or policy of, nor should any official endorsement be inferred on the part of, USUHS, the US Department of Defense, the US Department of Health and Human Services, the US Government, the Henry M. Jackson Foundation for the Advancement of Military Medicine, Inc, Guidehouse Inc, or Columbia University.

## Conflict of Interest

All authors have affirmed they have no conflicts of interest to declare.
